# Maternal overweight and obesity: a survey of clinicians’ characteristics and attitudes, and their responses to their pregnant clients

**DOI:** 10.1186/1471-2393-13-117

**Published:** 2013-05-21

**Authors:** Shelley A Wilkinson, Di Poad, Helen Stapleton

**Affiliations:** 1 Mater Research, Raymond Terrace, South Brisbane, Queensland 4101, Australia; 2 Mater Mothers’ Hospital, Raymond Terrace, South Brisbane, Queensland 4101, Australia; 3 Australian Catholic University, 1100 Nudgee Road, Banyo, QLD 4014, Australia

**Keywords:** Maternity, Guidelines, Midwifery, Obesity, Obstetrics, Staff characteristics, Weight perception, Fetal programming

## Abstract

**Background:**

Statewide (Queensland) Clinical Guidelines reflecting current best practice have recently become available for the management of pregnancy-related obesity. Our aim was to assess staff knowledge about, adherence to, and characteristics that influence delivery of care according to these Guidelines.

**Methods:**

An online survey, available over a three week period (May-June 2011), was disseminated to obstetric, midwifery and allied health staff working in a tertiary maternity hospital. Outcomes included knowledge of guideline content, advice given, knowledge of obesity pregnancy-related complications, previous training, referral patterns, and staff characteristics, including lifestyle habits, body satisfaction, and Body Mass Index (BMI).

**Results:**

Seventy-three staff completed surveys (59.6% response rate). Mean self-reported BMI was 24.2 ± 4.1 kg/m^2^ (17.9-36.4); 28.5% of staff were overweight (19%) or obese (9.5%), and 27.4% were underweight. However, 28.6%, 2.4%, and 1.2% ‘self-classified’ themselves as overweight, obese, and underweight, respectively. Almost 40% were dissatisfied/extremely dissatisfied with their weight. While the majority reported overweight/obesity (ow/ob) as an important/very important general obstetric issue and most correctly identified associated perinatal complications, only 32.1% were aware of existing guidelines, with only half correctly identifying BMI categories for ow/ob. A quarter indicated they did not provide women with gestational weight gain (GWG) advice relative to BMI category. Staff identified they would like more training in the area of supporting women to achieve and understand the need for healthy GWG. Staff role was significantly associated with guideline adherence (*p=0.03*) and association with BMI category approached significance (*p=0.07*). An association was observed between staff’s BMI and their belief in the influence of their advice on women’s GWG (*p=0.013*) and weight satisfaction and belief in women having the resources to make the changes they recommend (*p=0.003)*.

**Conclusions:**

Whilst lack of guideline knowledge provides a barrier to best-practice care, our findings suggest an interplay between staff confidence and personal characteristics in delivering such care which deserves recognition in staff education and training, and service development programs and future research.

## Background

Prevalence statistics for obesity suggest it is now pandemic, affecting populations across the age span, wealthy and impoverished nations alike, and without regard to traditional rural/urban divides [1]. Australia is one of the most severely affected developed countries [2], with more than 50% of Queensland adults weighing more than is recommended for good health [3]. The economic impact of inaction, or lack of effective action, is likely to be substantial as nations seek to manage the diverse health-related consequences against ever-increasing health-care costs [4]. In Australia these costs were estimated at approximately $10billion in 2008 (an increase of $7billion from 2005) [5], whilst in the United Kingdom (UK) costs are predicted to be at least £46 billion/year by 2050 [6].

The seemingly inexorable rise in infant and childhood obesity, associated with life-threatening co-morbidities such as early onset of type 2 diabetes mellitus and cardiovascular disease, has raised concerns that current and future generations of children may be the first to die before their parents [7]. Women of childbearing age, and especially those who are pregnant, must be considered a priority population for intervention, not least because pregnancy presents a ‘window of opportunity’ to reduce the burden of lifetime disease. Maternal diet and lifestyle factors affect fetal programming and hence influence pregnancy outcomes, including increasing the risk to the infant of developing (obesity-related) chronic diseases [8]. Furthermore women, as mothers, continue to play a central and traditional role in food provision and are thus influential in protecting their children against obesity and associated morbidities.

Statewide (Queensland) Clinical Guidelines reflecting current best practice have recently become available for the management of pregnancy-related obesity [9]. They provide advice regarding recommended gestational weight gain (GWG) based on pre-pregnancy body mass index (BMI), referral practices for multidisciplinary care including specialist support, and advice for the postnatal period, with demonstrated links to improved maternal and infant outcomes. Diet quality decreases with higher BMI categories [10] and women who start pregnancy in the overweight or obese range are at higher risk of metabolic complications (gestational diabetes, gestational hypertension and pre-eclampsia) and assisted deliveries, and their infants are at higher risk of macrosomia, structural birth defects, perinatal death, and becoming obese in childhood [8].

Despite the increased availability of evidence-based information describing obesity-associated complications, maternity professionals do not necessarily incorporate this into their interactions with pregnant women [11,12]. Clinician advice which is discordant with that provided in clinical Guidelines has also been reported [13,14]. Research further suggests that lack of confidence with initiating ‘difficult’ conversations about healthy weight management and fear of causing offence, are additional barriers to clinicians engaging with this issue [15-19]. Furthermore, even when maternity staff discuss appropriate GWG with pregnant women, a recent study in an Australian setting revealed that many considered the training they had received was inadequate; unfamiliarity with relevant Guidelines was a significant conversation barrier [20]. A reluctance by health professionals to engage in behaviour change discussions with patients, but rather reassigning this as “not my job” confirms this attitude as a major impedance to improving health outcomes [18].

Our research supports the premise of Herring et al. (2010), who suggested that personal characteristics, including body satisfaction and confidence, and lifestyle factors such as participating in regular physical activity, may also play an important role in predicting clinicians’ compliance with guidelines and their propensity to engage in weight management strategies with their pregnant clients [12]. Over 30% of pregnant women currently accessing our public tertiary maternity hospital in South East Queensland (with ~ 5,000 births per annum) are overweight or obese (ow/ob) and previous research has indicated that many would like advice and support around this issue [21]. Although American studies have assessed obstetric provider recommendations and knowledge [12,16], we could find no studies in this area which assessed aspects of maternity professionals’ lifestyles and personal characteristics against selected elements of clinical practice. Our study aimed to assess this deficit.

## Methods

### Participants

Obstetric, midwifery and allied health staff working in a tertiary maternity hospital were invited, via an email sent by their managers, to complete an adapted online survey [12] which was available for three weeks; completion required approximately 20 minutes. An information sheet was attached to the email invitation and two reminders were sent at the end of the first and second weeks respectively; the final reminder restated the cut-off date for completion. As participation in the survey was voluntary, consent was implied by completion of the survey; consent forms were not issued and the online platform rendered all responses anonymous. Staff who completed the survey were offered the opportunity to anonymously enter a draw to win one of three $50 department store vouchers.

### The survey tool

The survey requested professional and personal characteristics from staff, including profession, years of practice, previous training in the care of ow/ob pregnant women, gender, age, lifestyle habits including physical activity undertaken which we compared with the National Physical Activity Guidelines [22], whether staff were dieting or exercising to lose weight, body weight satisfaction, and BMI. We also assessed staff awareness of pregnancy-related ow/ob and associated complications, and their knowledge and application of the Statewide Obesity Guidelines [9]. Practice-based questions requested information about the clinical care staff provided and their opinions about their patients’ abilities to manage their GWG and/or change their lifestyles and behaviours to improve pregnancy outcomes. Additional information was sought about whether staff thought the advice they offered was helpful and/or if they considered that it influenced patient motivation and behaviour, including willingness to instigate change; referral patterns to services such as dietetics and anaesthesiology were also assessed. Staff were asked to comment on the resources available to them, including whether they had sufficient time and knowledge to offer counselling/advice. They were also asked to describe any specialist training they had received to assist them in working with this client group and whether they would like additional training and/or on-going inservice education. Although the aforementioned Statewide Guideline focuses on obesity, due to the significant, and increasing, proportion of the maternity population with BMIs above 25 kg/m^2^ and the clear evidence that this cohort are at risk of excessive GWG [23,24], we also invited commentary on overweight status as a likely prelude to obesity.

### Data analysis

Data were analysed using SPSS for Windows version 15 (SPSS, Chicago, Illinois). Descriptive statistics were used to summarise staff characteristics. Means and standard deviations were used to summarise normally distributed continuous data. Categorical data were summarised using frequencies and percentages. A composite guideline adherence score was calculated by summing responses to questions adapted from the 12 key recommendations in the Statewide guideline as ‘correct’ or ‘incorrect’. We expanded the 12 recommendations to 15, splitting those with double responses (e.g. ‘Offer diet and physical activity counselling to women’ was split to: i. ‘Offer diet counselling to women’ and ii. ‘Offer physical activity counselling to women’). Staff rated their responses on a four point Likert scale from ‘strongly disagree’ to ‘strongly agree’.

Exploratory data analysis investigated the association between guideline adherence and staff characteristics (ANOVA – categorical; linear regression – continuous variables), BMI status and staff’s practice opinions (ANOVA), and weight satisfaction and staff’s practice opinions (correlation). Significance was set at p<0.05.

Ethical approval was obtained from the Mater Health Services Human Research Ethics Committee and Griffith University Human Research Ethics Committee. The study was not funded and was conducted in the authors’ own time.

## Results

Seventy three staff completed surveys (overall 59.6% response rate). This comprised: 20/40 obstetrics(29.4%), 35/58 midwifery (51.5%) and 13/16 allied health (19.1%) staff. All staff provided their weight and height, and mean (calculated) BMI was 24.2 ± 4.1 kg/m^2^ (17.9-36.4). Almost one third (28.5%) of staff were overweight (19%) or obese (9.5%), and 27.4% were underweight. However, 28.6%, 2.4%, and 1.2% self-classified themselves as overweight, obese, and underweight, respectively, and over 21.4% did not answer this question. Almost 40% were dissatisfied or extremely dissatisfied (32.1% and 7.1%, respectively) with their current weight. Almost two-thirds (61.9%) reported a current stable weight, with13.1% decreasing, and 2.4% increasing, weight (22.6% missing). Just over one fifth (21.4%) were dieting to lose weight (missing 21.4%, n = 18) and 27.4% were exercising to lose weight (22.6%, n = 19). Over half (56%) of staff met the National Physical Activitiy Guidelines [22]. Two-thirds (67.9%) of respondents had never smoked, 8.3% had quit over a year ago, 2.4% had quit within the previous year, and 1.2% identified as currently smokers (20.2% missing, n = 17). Staff characteristics are displayed in Table 1.

**Table 1:** Characteristics of maternity care providers

		**Provider type**			
**Staff characteristics**	**Total %(n)**	**Obstetrics**	**Midwifery**	**Physiotherapy**	**Dietetics**
**Female**	**68 (81)**	**60%**	**91.4%**	**100%**	**100%**
**Responses from eligible staff**	**68/114 (59.6)**	**40 staff**	**58 staff**	**13 staff**	**3 staff**
		**20 (29.4)**	**35 (51.5)**	**10 (14.7)**	**3 (4.4)**
		**[Consultant n=6, Registrar n=6,Resident n=8]**			
**BMI (kg/m**^**2**^**), calculated from self-reported weight and height**					
Underweight	23 (27.4)	2 (10)	12 (34.3)	0 (0)	0 (0)
Healthy Weight	37 (44.0)	11 (55)	12 (34.3)	8 (80)	3 (100)
Overweight	16 (19)	3 (15)	9 (25.7)	1 (10)	0 (0)
Obese	8 (9.5)	4 (20)	2 (5.7)	1 (10)	0 (0)
Missing	0 (0)	0 (0)	0 (0)	0 (0)	
**Self-perceived BMI status (kg/m**^**2**^**)**					
Underweight	1.2 (1.2)	1 (5)	0 (0)	0 (0)	0 (0)
Healthy Weight	39 (46.4)	10 (50)	14 (40.0)	7 (70)	3 (100)
Overweight	24 (28.6)	8 (40)	8 (22.9)	3 (30)	0 (0)
Obese	2 (2.4)	0 (0)	2 (5.7)	0 (0)	0 (0)
Missing	(21.4)	1 (5)	11 (31.4)	0 (0)	0 (0)
**Satisfaction with body weight**					
Extremely dissatisfied	6 (7.1)	2 (10)	2 (5.7)	0 (0)	0 (0)
Dissatisfied	27 (32.1)	7 (35)	12 (34.3)	4 (40)	0 (0)
Neither	18 (21.4)	6 (30)	4 (11.4)	3 (30)	3 (100)
Somewhat satisfied	15 (11.9	3 (15)	3 (8.6)	3 (30)	0 (0)
Very satisfied	5 (6.0)	1 (5)	3 (8.6)	0 (0)	0 (0)
Missing	18 (21.4)	1 (5)	11 (31.4)	0 (0)	0 (0)
Undertaking sufficient* physical activity (yes)	47 (56)	14 (70)	15 (42.9)	7 (70)	2 (66.7)
Missing	21 (25)	0 (0)	0 (0)	1 (10)	0 (0)
**Undertaking exercise to lose weight (yes)**	23 (27.4)	4 (20)	8 (22.9)	6 (60)	0 (0)
Missing	19 (22.6)	1 (5)	11 (31.4)	1 (10)	0 (0)
**Dieting to lose weight (yes)**	18 (21.4)	3 (15)	6 (17.1)	5 (50)	0 (0)
Missing	18 (21.4)	1 (5)	11 (31.4)	5 (50)	0 (0)

*as defined by the National Phyisical Activity Guidelines.

The majority (88.1%) of staff reported ow/ob as an important/very important maternity health issue, with 76.2% indicating it was important/very important in their own clinical practice. Whilst most staff correctly identified associated complications, only 32.1% were aware of the Statewide Guidelines. Just under 50% of staff correctly identified BMI categories for ow/ob (Table 2). Few (<8%) identified appropriate GWG goals and 25% indicated they did not provide their maternity patients, including those who are ow/ob, with any advice on the issue of healthy GWG relative to BMI category. When specifically questioned about reasons for not providing dietary advice, 19% of staff reported that they lacked the requisite background and/or knowledge in this area, 15.5% reported having no time and 11.9% that it was not their job. A further 10.7% did not feel comfortable discussing dietary changes, and 3.6% felt the woman would not make the changes they suggested. Survey results indicated that only 20.2% of staff had received training in providing care for ow/ob pregnant women.

**Table 2:** Proportion of correct responses to questions regarding BMI categories and pregnancy

**BMI category and gestational weight gain goal**	**Proportion of staff who *correctly identified cut-offs *for each BMI range (%)**	**Proportion of staff who *correctly identified the proportion *of women in each BMI category (%)**	**Proportion of staff who *provided correct weight gain advice*, per BMI category (%)**
**Underweight** (<18.5 kg/m^2^: 12½ -18 kg)	7.1	20.2	3.6
**Healthy weight** (18.5-24.9 kg/m^2^: 11½ -16 kg)	11.9	20.2	6.0
**Overweight** (25–29.9 kg/m^2^: 7-11½ kg)	45.2	9.5	8.3
**Obese** (>30 kg/m^2^:5-9 kg)	50.2	21.4	7.1

Adherence to recommendations for referral was variable; only 45.5%, 12.8%, and 5.4% correctly identified the Guideline-defined BMI cut-off for referral to anaesthetics, to dietetics, and an early oral glucose tolerance test (OGTT), respectively. Over ten percent (14.5%) of staff considered referrals to anaesthetists was ‘not their job’, to dietitians (20%) and for an OGTT (20%). Staff from all groups valued being able to refer women to a dietitian and felt that women were able to change, although midwives appeared less convinced in both areas compared with other professional groups. Staff perceptions about the influence of their advice, and the time available for supporting women who may not have the resources to sustain lifestyle changes to manage GWG, were rated more moderately. Staff also identified they would like more training in the area of supporting women to achieve healthy GWG.

A positive association was observed between staff’s BMI and weight satisfaction and staff practice opinions and behaviours (Table 3). Staff’s BMI was significantly associated with staff ratings of their belief in their advice to influence women’s GWG; higher BMIs were associated with greater staff ratings of ability to influence women (*ß = 0.5, p = 0.013*). Staff’s satisfaction with their own weight was significantly correlated with ratings regarding their belief that women have the necessary resources to make the recommended changes; higher weight satisfaction was correlated with higher ratings regarding women’s ability to follow recommended changes (*r = 0.54, p = 0.003*) (Table 3).

**Table 3:** Associations between staff practices and staff characteristics (BMI, weight satisfaction)

	**BMI**	**Weight satisfaction**
My advice influences how much weight my patients gain during pregnancy	β = −0.5, *p=0.013*	r =0.2, *p= 0.5*
I have enough time to counsel my patients properly about the risks of overweight/obesity during pregnancy	β = −0.3, *p=0.071*	r =0.02, *p=0.9*
I have sufficient knowledge to counsel my overweight/obese patients to improve their pregnancy outcomes	β = −0.2, *p = 0.3*	r =0.002, *p=1.0*
I would like more training about to help me counsel my overweight/obese patients to improve their pregnancy outcomes	β =0.02, *p=0.9*	r =−0.2. *p=0.3*
Nutrition and dietetic referrals are available for my overweight/obese patients	β =0.007, *p=1.0*	r =0.1, *p=0.5*
Once an overweight/obese woman is already pregnant, there is not much that she can do to change the risks of poor outcomes	β =0.04, *p=0.8*	r =0.1, *p=0.5*
My overweight/obese patients:		
• are motivated to make changes to their health	β =−0.009, *p =1.0*	r =−0.1, *p = 0.5*
• have the resources they need to make the changes that I recommend	β =−0.1, *p=0.6*	r =0.54, *p = 0.003*
• find my advice helpful regarding weight management during pregnancy	β =0.009, *p= 1.0*	r =−0.1, *p = 0.7*

Adherence to Guideline recommendations was rated out of 15 with scores from the four different professional groups of respondents, ranging from 3.8 ± 2.0 to 10.3 ± 0.6 (Physiotherapy 3.8 ± 3.6; Midwifery 6.3 ± 4.2; Obstetrics 6.3 ± 2.7; Dietetics 10.3 ± 0.6, *p = 0.03*) (Table 4). No other variable was significantly associated with guideline adherence, although BMI category approached significance (*p=0.07*). The association between BMI status (as a continuous variable) and guideline adherence also approached significance (*p=0.06*). Significant differences were seen between guideline adherence and whether staff offered weight gain advice never (2.5 ± 2.6), sometimes (6.0 ± 3.1) or always (8.8 ± 2.7), *p<0.001.*

**Table 4:** Relationship between staff variables and adherence to Queensland Health Obesity guidelines

**Variables**		**Guideline score (/15)**	**Level of significance**
Gender	Male	6.8 ± 3.8	*p=0.6*
	Female	6.1 ± 3.5	
Profession	Obstetrics	6.3 ± 2.7	*p=0.03*
	Midwifery	6.3 ± 4.2	
	Dietetics	10.3 ± 0.6	
	Physiotherapy	3.8 ± 3.6	
Years in role	<1	5.7 ± 3.4	*p=0.2*
	1-5	5.4 ± 3.1	
	6-10	8.6 ± 4.5	
	11-20	6.5 ± 3.1	
	>20	5.3 ± 4.0	
Attended training in the care of overweight/obese maternity patient	Yes	7.1 ± 2.8	*p=0.3*
	No	5.8 ± 3.7	
BMI	Underweight	4.1 ± 3.1	*p=0.07*
	Healthy Weight	5.6 ± 3.6	
	Overweight	7.8 ± 3.1	
	Obese	7.3 ± 3.5	
Weight satisfaction	Very dissatisfied /dissatisfied	6.5 ± 3.4	*p=0.5*
	Neutral	6.9 ± 3.8	
	Very satisfied/satisfied	5.4 ± 2.6	
Meets physical activity guidelines	Yes	6.5 ± 3.6	*p=0.9*
	No	6.4 ± 3.5	

## Discussion

This study assessed how aspects of maternity professionals’ lifestyles and personal characteristics potentially influenced their consultations with pregnant ow/ob women. Although the groups surveyed (midwives, obstetricians, dietitians, physiotherapists) were able to cite many negative perinatal outcomes associated with maternal ow/ob status, compliance with referral guidelines and recommended advice to ow/ob pregnant women was poor. This reiterates findings from a recent American study [12]. A possible correlation between health professionals’ BMI status and other personal characteristics and lifestyle preferences, and their knowledge and willingness to engage with ow/ob women about strategies to manage GWG, needs futher investigation. Our research findings also reflect other studies in this area [12,16,18].

Failure to adopt the Statewide Guidelines is a concern. However, we acknowledge that guidelines alone are rarely sufficient to effect changes in clinical practice, as successful translation requires clear implementation strategies, based on systematically identified gaps or barriers that may include knowledge, but also staff skills, workplace systems, and team dynamics [25]. With respect to translation in the context of our hospital, this will include a strong focus on staff training to remedy the knowledge gaps and skills deficits identified in our survey. A more detailed strategy is outlined in our recent publication [26]. Planned interventions will also address the broader systems and policy changes required to maximise team functioning and approaches to service delivery [14,27,28], including the re-introduction of the importance of monitoring weight during pregnancy [29].

Generally low awareness was found across all professional groups surveyed (aside from dietetics) about BMI cut-off ranges, and associated GWG advice for ow/ob women, which has been recognised in another recent Australian study [20]. This is of some concern as the evidence strongly suggests that if pregnant women are not advised about appropriate GWG they are more likely to gain outside the recommended range [30]. Hence, the onus of responsibility on maternity professionals to ensure appropriately timed, evidence-based, information is disseminated to their clients, especially those who are ow/ob at the outset of pregnancy as they are most at risk of excess GWG. Our findings hint at a link between knowledge deficits amongst staff regarding BMI categories for underweight/overweight/obese, and their individual BMI status and body (dis)satisfaction, however this requires further research. The impact of shifting social norms, including the ‘creeping normality’ and wider acceptance of ow/ob (as evidenced, for example the by International ‘Fat Activist’ movement), and a failure to recognise obesity as a serious and chronic condition, also needs further investigation [31-34].

The tendency for health professionals to compartmentalise their role and impose limits on their clinical responsibilities, sometimes in response to perceived pressures on their time and/or inadequate resources, has been reported elsewhere [18]. An additional feature we observed was a tendency for midwives to regard to regard themselves as something of a ‘one-stop-shop’, insofar as they appeared reluctant to refer women, but rather assumed responsibility for all aspects of care provision. This may be because midwives are typically more likely than other maternity colleagues to have sustained contact with women over the duration of pregnancy. Guidelines strongly suggest, however, that multidisciplinarity is a key feature of a successful service, especially for clients with complex needs [9].

Critics have argued that the rapid, and continued, increase in obesity has left maternity services and staff inadequately prepared to meet the needs of affected women; midwives in particular have been described as “not waving but drowning” [31] in their attempts to access appropriate resources. Recent research has also highlighted confusion and uncertainty amongst maternity professionals regarding the most appropriate approach to take when counselling their obese patients, with concerns articulated about being effective but not offensive, in the way they convey information [16,17].

Societal stigma associated with obesity has relevance for all health professionals striving to maintain good patient relationships, whilst providing evidence-based care [18]. Gray et al’s (2011) assertion that “words matter” is a reminder that terminology acceptable to patients may differ from that routinely used by health professionals [35] with words such as ‘weight’ and ‘BMI’ more likely to be positively received than references to ‘fat’ and/or (morbidly) ‘obese’ [36,37]. The stigmatisation traditionally associated with excessive weight is increasingly challenged, however, by what has been referred to as the “creeping normality” [38] and greater acceptance of ow/ob as the proportion of affected people continues to steadily increase worldwide. Indeed, people considered to be of normal weight, with healthy BMIs, are already in a minority in selected populations [39,40], with experts warning that this trend seems likely to continue [41].

When advising pregnant women about healthy weight gain goals (which are informed by pre-pregnancy BMI [29]), staff are advised to avoid direct reference to BMI categories (e.g. ‘underweight’, ‘overweight’, ‘obese’ etc). Within our local context, anecdotal reports suggest that the phrase *‘based on your pre-pregnancy weight, you should aim to gain xx-xx kg for the healthiest pregnancy possible’* is well received by pregnant women. Skills-based training, contextualised locally and developed to meet the specific needs of health professionals, and which is based on proven theoretical models such as behaviour change theory, is likely to produce better results than training that relies on strategies which, although perhaps widely used, are not evidence-based [18,19].

A strength of our research is that, to the best of our knowledge, it is the first Australian study to examine maternity professionals’ knowledge, attitudes, practices, and lifestyle behaviours, in conjunction with a clinical guideline and maternity care provided to ow/ob pregnant women. Although our response rate was lower than desirable, it was similar to that of Herring et al. [12] and may be partly explained by the route (email) selected to deliver the survey link. The less than optimal response rate may also be accounted for by the fact that many staff are employed on a part-time or rotational basis, which makes effective contact difficult to establish and maintain. A further limitation of our study, also noted by Herring et al. [12], was that we used an unweighted guideline adherence score which assumed all components have equal importance in care delivery to this patient population, whereas in fact they may not.

We anticipate that our findings will help to inform the development and delivery of tailored training programs for different staff groups working within the maternity service of our large tertiary unit. While we are an urban-based hospital providing care to ‘low risk’ pregnant women as well as those with complex needs, and the general health profile of our staff was higher than the general population (as suggested by a lower prevalence of smoking, BMIs ≥ 25 kg/m^2^ and a greater proportion meeting physical activity guidelines) [42], we nonetheless anticipate that discrete elements of our findings will be applicable to other maternity environments with more varied staff profiles.

Effecting the changes outlined above is likely to exceed the managerial and clinical scope of individual practitioners and will require Institutional commitment, with clear directives from senior executives, and an organisational culture which recognises the imperative of evidence-based practice. It also requires an ongoing commitment to ensuring staff are well-trained and resourced, and up to date with current guidelines and policy recommendations.

## Conclusion

In conclusion, although a Statewide (Queensland) Guideline exists and is intended to inform care delivery for pregnancy-related obesity, staff responses to our survey indicated a marked discordance with Guideline content. Whilst we recognise that lack of knowledge may inhibit the delivery of best practice, our findings suggest a more complex interplay between staff confidence and personal characteristics in the delivery of care, as suggested by the significant number of front-line staff across the four professional groups who were themselves dissatisfied with their own weight and body image. We suggest that further research is needed to identify whether, and to what extent, clinicians’ personal characteristics and lifestyle habits might be a factor in poor guideline compliance and a barrier to counselling ow/ob women effectively, and referring them appropriately.

## Competing interests

The authors declare that they have no competing interests.

## Authors' contributions

SW was involved in the planning of this project, the collection, analysis, and interpretation of data, and the writing of the manuscript. HS was involved in project planning, data collection, analysis and interpretation of the data, as well as manuscript preparation. DP was involved in interpretation of the data and manuscript preparation. All authors read and approved the final manuscript.

## Pre-publication history

The pre-publication history for this paper can be accessed here:

http://www.biomedcentral.com/1471-2393/13/117/prepub
